# *GJB4* variants linked to skin disease exhibit a trafficking deficiency en route to gap junction formation that can be restored by co-expression of select connexins

**DOI:** 10.3389/fcell.2023.1073805

**Published:** 2023-02-13

**Authors:** Sergiu A. Lucaciu, Rhett Figliuzzi, Ruth Neumann, Samina Nazarali, Luigi Del Sordo, Stephanie E. Leighton, Alexandra Hauser, Qing Shao, Danielle Johnston, Donglin Bai, Dale W. Laird

**Affiliations:** ^1^ Department of Anatomy and Cell Biology, University of Western Ontario, London, ON, Canada; ^2^ Department of Physiology and Pharmacology, University of Western Ontario, London, ON, Canada

**Keywords:** connexin, epidermis, keratinocyte, trafficking, connexin 30.3 (Cx30.3), gap junctional intercellular communication

## Abstract

Epidermal keratinocytes are enriched with at least nine connexins that are key regulators of epidermal homeostasis. The role of Cx30.3 in keratinocytes and epidermal health became evident when fourteen autosomal dominant mutations in the Cx30.3-encoding *GJB4* gene were linked to a rare and incurable skin disorder called erythrokeratodermia variabilis et progressiva (EKVP). While these variants are linked to EKVP, they remain largely uncharacterized hindering therapeutic options. In this study, we characterize the expression and functional status of three EKVP-linked Cx30.3 mutants (G12D, T85P, and F189Y) in tissue-relevant and differentiation-competent rat epidermal keratinocytes. We found that GFP-tagged Cx30.3 mutants were non-functional likely due to their impaired trafficking and primary entrapment within the endoplasmic reticulum (ER). However, all mutants failed to increase BiP/GRP78 levels suggesting they were not inducing an unfolded protein response. FLAG-tagged Cx30.3 mutants were also trafficking impaired yet occasionally exhibited some capacity to assemble into gap junctions. The pathological impact of these mutants may extend beyond their trafficking deficiencies as keratinocytes expressing FLAG-tagged Cx30.3 mutants exhibited increased propidium iodide uptake in the absence of divalent cations. Attempts to rescue the delivery of trafficking impaired GFP-tagged Cx30.3 mutants into gap junctions by chemical chaperone treatment were ineffective. However, co-expression of wild type Cx30.3 greatly enhanced the assembly of Cx30.3 mutants into gap junctions, although endogenous levels of Cx30.3 do not appear to prevent the skin pathology found in patients harboring these autosomal dominant mutations. In addition, a spectrum of connexin isoforms (Cx26, Cx30, and Cx43) exhibited the differential ability to trans-dominantly rescue the assembly of GFP-tagged Cx30.3 mutants into gap junctions suggesting a broad range of connexins found in keratinocytes may favourably interact with Cx30.3 mutants. We conclude that selective upregulation of compatible wild type connexins in keratinocytes may have potential therapeutic value in rescuing epidermal defects invoked by Cx30.3 EKVP-linked mutants.

## Introduction

Gap junctions are clusters of intercellular channels composed of connexins that establish a syncytium of gap junctional intercellular communication (GJIC) to allow the passage of numerous small molecules and metabolites between cells; an essential process for skin homeostasis and epidermal health (Richard, 2000; Srinivas et al., 2018; Laird and Lampe, 2022). Connexins (Cx) are a 21-member protein family that oligomerize into homomeric or heteromeric hexamers known as connexons (Söhl and Willecke, 2004; Laird, 2006; Goodenough and Paul, 2009; Lampe and Laird, 2022). At the cell surface, connexons can function independently as hemichannels or proceed to form a variety of homotypic or heterotypic intercellular channels that provide epidermal keratinocytes with a wide array of channel diversity (Cottrell and Burt, 2005; Koval, 2006; Meşe et al., 2007; Martin et al., 2014; Faniku et al., 2015; Bai et al., 2018). Under physiological conditions, connexin hemichannels are thought to adopt a primarily closed conformation to maintain ionic gradients and prevent the leak of cytoplasmic contents that commonly leads to cell death (Peng et al., 2022). Growing evidence suggests that the pathological opening of hemichannels underlies inflammatory disease and various connexin-linked inherited disorders (Delmar et al., 2018; Cocozzelli and White, 2019; Peng et al., 2022).

The epidermis is unique amongst connexin expressing tissues, as keratinocytes express at least nine different connexin isoforms (Cx26, Cx30, Cx30.3, Cx31, Cx31.1, Cx37, Cx40, Cx43, and Cx45), referred to in this report as “keratinocyte connexins” (Di et al., 2001; Meşe et al., 2007; Faniku et al., 2015; Au et al., 2020). Keratinocyte connexins are expressed in distinct and overlapping spatial and temporal patterns as keratinocytes undergo migration through the strata of the epidermis during terminal differentiation. For example, Cx43 is primarily expressed in the basal layer of the epidermis, with decreasing expression in the upper layers, whereas Cx30.3 is primarily expressed in the stratum granulosum, together with other connexins such as Cx26, Cx30, Cx31, and Cx45 (Cartlidge, 2000; Macari et al., 2000; Richard, 2000; Di et al., 2001; Langlois et al., 2007; Faniku et al., 2015). The differential expression of keratinocyte connexins is thought to play a key role in epidermal differentiation, proliferation, wound repair, as well as in maintaining epidermal homeostasis (Candi et al., 2005; Proksch et al., 2008; Churko and Laird, 2013; Martin et al., 2014; Faniku et al., 2015; Anderton and Alqudah, 2022).

The importance of keratinocyte connexins in the epidermis is best exemplified by the various skin diseases caused by connexin gene mutations. Mutations in five keratinocyte connexins, namely the β-connexins Cx26 (encoded by *GJB2*), Cx30 (encoded by *GJB6*), Cx30.3 (encoded by *GJB4*), and Cx31 (encoded by *GJB3*), and the α-connexin Cx43 (encoded by *GJA1*) are linked to congenital skin diseases in humans, ranging from mild to life-shortening (Scott et al., 2012; Lilly et al., 2016; Laird et al., 2017; Delmar et al., 2018; Laird and Lampe, 2022). For example, mutations to *GJB2* can cause Bart-Pumphrey syndrome, and the more severe keratitis-ichthyosis-deafness syndrome, whereas mutations to *GJB6* are associated with Clouston syndrome (Richard, 2005). Mutations to *GJB3, GJB4*, and *GJA1* are linked to erythrokeratodermia variabilis et progressiva (EKVP), a disease characterized by hyperkeratotic plaques and erythematous patches (Ishida-Yamamoto, 2016).

Of the three connexins linked to EKVP, the mechanisms that underpin *GJB4* gene mutations (OMIM #617524) to disease remain largely unknown. It has been known for some time that wild type Cx30.3 assembles into functional gap junctions when expressed in *Xenopus* oocytes, but this process appears inefficient as Cx30.3 is also frequently found within intracellular compartments (Hennemann et al., 1992; Plantard et al., 2003; Zhang et al., 2021). Cumulatively, *GJB4* (Cx30.3) is the most common connexin gene linked to EKVP, with fourteen *GJB4* gene variants encoding thirteen distinct Cx30.3 mutant proteins. Most of these gene variants are subject to autosomal dominant inheritance with the notable exception of the Thr130Met (T130M) variant, where the mode of inheritance is unclear (Macari et al., 2000; Richard et al., 2003; Common et al., 2005; Scott et al., 2011; Kokotas et al., 2012; Sbidian et al., 2013; Dai et al., 2020; Zhang et al., 2021). Of these gene variants, only the Phe137Leu (F137L) and Val37Met (V37M) Cx30.3 mutants have been partially characterized (Plantard et al., 2003; Zhang et al., 2021). The myc-tagged F137L mutant was found to have a trans-dominant negative effect on the trafficking, gap junction assembly, and function of Cx31 when co-expressed in HeLa cells (Plantard et al., 2003), while reduced combined levels of Cx30.3 and the V37M mutant were noted in the epidermis of the patient harbouring the V37M mutant compared to healthy controls (Zhang et al., 2021). Furthermore, the V37M mutant also exhibited reduced expression and impaired trafficking *in vitro* when expressed in HeLa cells (Zhang et al., 2021). The trafficking and functional status of the remaining EKVP-linked Cx30.3 variants are unknown including whether they exhibit any “loss-of-function” or “gain-of-function” mechanism(s) of action that manifest in disease (Kelly et al., 2015b). We propose that the characterization of EKVP-linked Cx30.3 mutants and the underpinning mechanisms involved is imperative for the future development of any targeted treatments.

In the present study, we characterize the expression, localization, and function of GFP and/or FLAG tagged wild type human Cx30.3 as well as Gly12Asp (G12D), Thr85Pro (T85P), and Phe189Tyr (F189Y) Cx30.3 mutants in tissue-relevant and differentiation-competent rat epidermal keratinocytes (REKs). We selected these three mutants, classified as pathogenic under the American College of Medical Genetics and Genomics guidelines (Richards et al., 2015), because these amino acid sites are highly conserved throughout the β-connexin subfamily suggesting their probable importance in establishing Cx30.3 structure and function. Collectively, our results indicate that the EKVP-linked mutants are trafficking impaired and retained primarily in the ER and Golgi apparatus, where they fail to invoke ER stress as defined by the lack of increased BiP/GRP78 levels. However, their pathological properties may extend beyond their trafficking deficiency as expression of Cx30.3 mutants was also found to increase propidium iodide uptake in the absence of extracellular divalent cations, which may reflect the loss of cell membrane integrity leading to cell death. While cellular conditions designed to improve protein folding and ER function failed to improve the trafficking of EKVP-linked mutants and gap junction formation, co-expression of wild type Cx30.3 dramatically increased mutant assembly into gap junctions while other co-expressed keratinocyte connexins all variably improved Cx30.3 mutant assembly.

## Materials and methods

### Cell culture

Rat epidermal keratinocytes (REKs) were originally obtained from Dr. Vincent Hascall (Cleveland Clinic, Cleveland, Ohio, United States). REKs are spontaneously immortal but retain the capacity to form an organotypic rat epidermis when grown in an air-liquid interface (Maher et al., 2005; Au et al., 2020). REKs express transcripts for eight connexins, but only express high levels of Cx43 protein with trace levels of Cx26 detectable in overgrown cells (Maher et al., 2005; Au et al., 2020). Therefore, Cx43-ablated REKs (Cx43KO REKs) were generated using CRISPR/Cas9 technology as described and characterized earlier (Au et al., 2020). REKs, Cx43KO REKs, and normal rat kidney (NRK) cells were grown in sterile T25 (Fisher Brand, Burlington, ON, Canada, Cat# FB012935) or T75 flasks (Fisher Brand, Cat# FBO12937) containing Dulbecco’s modified Eagle’s medium (DMEM; ThermoFisher, Burlington, ON, Canada, Cat# 11960) fortified with 10% (v/v) fetal bovine serum (FBS; ThermoFisher, Cat# 12483-020), 2 mM L-glutamine (ThermoFisher, Cat# 25030-081), and 100 U/mL penicillin/streptomycin (ThermoFisher, Cat# 15140-122). Cells were cultured at 5% CO_2_ and 37°C (unless otherwise stated) and subcultured when 80%–90% confluent into 35 mm plastic culture dishes or 35 mm 6-well plates using 0.25% (w/v) trypsin-ethylene-diamine-tetraacetic acid (Trypsin-EDTA; ThermoFisher, Cat# 25200056). Connexin-deficient Neuro-2a (N2a) cells were used for electrophysiology experiments and cultured under the same conditions outlined above.

### Plasmids

The human Cx30.3-BFP expression vector driven by a CMV promoter was custom ordered from VectorBuilder (Chicago, IL, United States). Employing *XhoI* and *HindIII* restriction sites, the BFP tag was replaced by fusing Cx30.3 in-frame to mox-GFP (AddGene, Watertown, MA, United States, Cat# 68070). Plasmids encoding GFP-tagged Cx30.3 mutants (G12D, T85P, and F189Y) were special ordered and purchased from NorClone Biotech Laboratories (London, ON, Canada) and confirmed by sequencing. Red fluorescent protein (RFP) tagged Cx30.3, Cx26, Cx30, and Cx43 were described earlier (Berger et al., 2014; Au et al., 2020; Beach et al., 2020). The myc-DDK (FLAG)-tagged Cx30.3 expression vector driven by a CMV promoter was purchased from Origene (Rockville, MD, United States, Cat# RC204406). This plasmid was outsourced to NorClone Biotech Laboratories (London, ON, Canada) for the generation of the G12D, T85P, and F189Y mutants *via* site-directed mutagenesis. Resulting plasmids encoding Cx30.3 mutants were verified by sequencing.

### Transfection

REKs and Cx43KO REKs were cultured and grown to ∼50%–60% confluence and transiently transfected by mixing 0.1–1 μg of DNA constructs encoding GFP- or FLAG-tagged Cx30.3, G12D, T85P, and F189Y and 2 μl Lipofectamine 2000 (ThermoFisher, Cat# 11668019) or Lipofectamine 3000 (ThermoFisher, Cat# L3000015) in 200 μl of Opti-Mem reduced serum (ThermoFisher, Cat# 31985-070). This mixture was gently mixed and incubated at room temperature for 20 min (for Lipofectamine 2000) or 5 min (for Lipofectamine 3000) before being added dropwise to the REKs. In some cases, REKs were co-transfected with 1 μg of GFP-tagged Cx30.3 or Cx30.3 mutants with 1 μg of constructs encoding Cx30.3-RFP, Cx26-RFP, Cx30-RFP or Cx43-RFP (Berger et al., 2014; Au et al., 2020). On occasion, REKs or Cx43KO REKs were transfected with a plasmid encoding Cx43-GFP as a control. All transfected cells were used in experiments 24–48 h later.

N2a cells used for electrophysiology experiments were cultured and grown to ∼50%–70% confluence and transiently transfected by combining separate tubes containing 1) 1 μg of DNA constructs encoding GFP-tagged Cx30.3, G12D, T85P, F189Y, and 2) free GFP in 400 μl of Opti-Mem, and 2 μl X-tremeGENE HP DNA transfection reagent (MilliporeSigma, Oakville, ON, Canada Cat# 6366244001) in 400 μl of Opti-Mem and incubated at room temperature for 30 min (Yue et al., 2021; Jaradat et al., 2022). Growth media was aspirated, and cells were incubated with the transfection cocktail for 5 h at 37°C. Subsequently, the transfection cocktail was replaced with fresh serum-containing culture media and cells grown at 37°C and 5% CO_2_ overnight and used for electrophysiology experiments the following day as outlined below.

### Immunolabeling and imaging

Control cells or cells expressing GFP- or FLAG-tagged Cx30.3, Cx30.3 mutants or co-expressing Cx30.3 mutants together with RFP-tagged Cx30.3, Cx26, Cx30, or Cx43 were plated on sterile glass coverslips (ThermoFisher, Cat# 1254580). Cells were grown to ∼80% confluence, rinsed twice with 1x phosphate-buffered saline (PBS) (ThermoFisher, Cat# 10010-023) and fixed in ice-cold 80% methanol/20% acetone (v/v) solution for 15 min at 4°C. After rinsing in PBS, cells were blocked with 2% (w/v) bovine serum albumin (BSA; MilliporeSigma, Cat# A2153) for 30 min at room temperature. Cells were labelled for 45 min at room temperature with a 1:200 dilution of the following primary antibodies: mouse monoclonal anti-FLAG (MilliporeSigma, Cat# F3165), anti-PDI (Enzo Life Science, Cat# ADI-SPA-891F), anti-E-cadherin (BD Biosciences, Cat# 610182) or rabbit monoclonal anti-Cx43 (MilliporeSigma, Cat# C2619), anti-FLAG (Cell Signaling, Cat# 14793) and anti-BiP/GRP78 (MilliporeSigma, Cat# G8918). Cells were then washed with PBS and incubated for 45 min in the secondary antibody Alexa Fluor 555-conjugated goat monoclonal anti-mouse (ThermoFisher, Cat# A32727) or anti-rabbit (ThermoFisher, Cat# A32732) IgG (1:800 dilution in 2% BSA), or in some cases, AlexaFluor 488-conjugated donkey anti-mouse (ThermoFisher, Cat# A21202) or donkey anti-rabbit (ThermoFisher, Cat# A21206) IgG (both used at 1:800 dilution). All antibodies were diluted as indicated in blocking solution. All coverslips were then stained with Hoechst 33342 (1:1000 dilution in distilled water; ThermoFisher, Cat# H3570) for 1 min to demarcate the nuclei and mounted onto glass microscope slides using Airvol mounting solution. In some cases, cells were only labelled with Hoechst 33342.

Cells were imaged on a Zeiss LSM800 confocal microscope equipped with Zen2.3 software (Zeiss International, Oberkochen, Germany, www.zeiss.com) and a ×63 oil immersion objective lens. When imaging samples for direct comparisons, all images were acquired and presented using identical imaging conditions (laser strength, pinhole, and contrast) to ensure fluorescence intensity could be directly compared. For gap junction quantification, a third-party investigator blinded to the treatment quantified the percent of gap junction forming cell pairs. Gap junction plaques were defined as a linear or punctate green fluorescence signal of approximately 0.2 μm in length, situated at the interface between two transfected cells. Experiments were repeated at least three times, with 30 or more cells or cell-cell interfaces analyzed for each group in each repeat.

### Multi sequence alignment and generation of connexin sequence logos

The OMA (Ortholog MAtrix) phylogenomic database (https://omabrowser.org/) was queried for the available primary sequences of Cx30.3 and all seven β-connexins from various vertebrate species. Multi sequence alignment was then performed using Clustal Omega in Jalview version 2.11.2.0 (https://jalview.org/). Sequence logos were then generated for the span of nine amino acids flanking the G12, T85, or F189 residues using the WebLogo sequence logo generator (https://weblogo.berkeley.edu/) (Schneider and Stephens, 1990; Crooks et al., 2004; Bai, 2016).

### Bioinformatics analysis

The pathogenicity of missense mutants was predicted using several algorithms, including SIFT (Kumar et al., 2009), SNPs & GO (Calabrese et al., 2009), MutPred2 (Pejaver et al., 2020), PolyPhen-2 (Adzhubei et al., 2010), and CADD (Rentzsch et al., 2021). The sequences of the missense mutants were input into the algorithms and compared against the wild type Cx30.3 reference sequence taken from UniProt (UniProt ID Q9NTQ9; http://uniprot.org) (Bateman et al., 2021). The genome aggregation database (gnomAD; https://gnomad.broadinstitute.org/) was also queried to determine whether the three mutants were common genetic polymorphisms (Karczewski et al., 2020). Allele frequencies for the G12D (allele ID 20046), T85P (allele ID 20045) and F189Y (allele ID 20048) were taken from the ClinVar database (https://www.ncbi.nlm.nih.gov/clinvar/).

### Western blotting

REKs engineered to transiently express FLAG-tagged Cx30.3 or Cx30.3 mutants were subjected to SDS-PAGE and Western blotting as previously described (Au et al., 2020; Lucaciu et al., 2022). Blots were labeled with rabbit anti-FLAG (1:3000) and mouse anti-GAPDH (1:5000; MilliporeSigma, Cat# MAB374) antibodies overnight at 4°C. Primary antibodies were removed, blots were washed, and then labeled with goat anti-mouse IgG AlexaFluor 680 (1:10000) (ThermoFisher, Cat# A-21057) and goat anti-rabbit IgG DyLight™ 800 (1:10000, ThermoFisher, Cat# SA5-10036) for 45 min at room temperature. Secondary antibodies were removed, blots washed, and protein bands were subsequently visualized using the LI-COR Odyssey infrared imaging system. In some cases, control and cells treated with 2 μg/mL of tunicamycin (MilliporeSigma, Cat# 11089-65-9) for 24 h or cells engineered to express Cx30.3 or mutants were immunoblotted for BiP/GRP78 (1:2000) to probe for UPR activation.

### Dual whole-cell patch clamp

Transiently transfected N2a cells were re-plated onto glass coverslips and incubated for 2 h at 37°C and 5% CO_2_. Coverslips were transferred to a recording chamber on a fluorescent microscope (Leica DM IRB) and subsequently washed and bathed in extracellular solution (140 mM NaCl, 2 mM CsCl, 2 mM CaCl_2_, 1 mM MgCl_2_, 5 mM HEPES, 4 mM KCl, 5 mM D-Glucose, 2 mM pyruvate, pH 7.4). Glass micropipettes were pulled with a PC-100 puller (Narishige, Amityville, NY, United States) configured to yield a patch pipette resistance in the range of 2–3 MΩ, and back-filled with intracellular solution (130 mM CsCl, 10 mM EGTA, 0.5 mM CaCl_2_, 3 mM MgATP, 2 mM Na_2_ATP, and 10 mM HEPES, pH 7.2). Isolated transfected cell pairs, identified *via* fluorescence from the covalently bound GFP tag, were selected for dual whole-cell patch clamp and a voltage clamp at 0 mV was applied to each cell. In an alternating fashion, one cell in the pair was held at a constant holding potential of 0 mV and junctional currents (*I*_*j*_) were recorded in this cell during the application of 7 s long trans-junctional voltage (*V*_*j*_) pulses (at ±20, ±60, and ±100 mV) to the other cell of the pair as previously described (Xin et al., 2010; Tong et al., 2014; Yue et al., 2021; Jaradat et al., 2022; Lucaciu et al., 2022). The *I*_*j*_ recorded during the ±20 mV *V*_*j*_ pulse was used to calculate the macroscopic junctional conductance (*G*_*j*_ = *I*_*j*_ ÷ *V*_*j*_) (Musa et al., 2004; Bai and Cameron, 2016).

### Fluorescence recovery after photobleaching (FRAP)

REKs were subcultured into 35 mm glass-bottom dishes (Matsunami, Cat# D1123OH) and transfected as described earlier. Cells were loaded with CellTrace™ Calcein Red-Orange, AM (5 μg/mL; ThermoFisher, Cat# C34851) dye solution diluted in 1 mL of OptiMEM reduced serum for 10 min at 37°C and 5% CO_2_. The cells were then washed three times with 1x PBS, covered with 2 mL of Opti-Mem and transferred to a live cell imaging apparatus on the Zeiss LSM800 confocal microscope heated to 37°C. A single cell in a small cluster or pair of transfected cells was selected and imaged with a ×63 oil immersion objective prior to photobleaching with the Zeiss LSM800 confocal microscope with Zen2.3 software. The selected fluorescent cells were photobleached to reduce the fluorescence by ∼80% intensity using a 561 nm laser, and fluorescence recovery was imaged every second for 2 min. Fluorescence recovery was quantified using the Time Series Analyzer v3 Plugin for ImageJ (NIH, imagej.nih.gov/ij/) as previously described (Au et al., 2020; Lucaciu et al., 2022). Fluorescence recovery was measured by dividing the fluorescence at each 1-s interval and the level of fluorescence immediately after photobleaching by the initial fluorescence intensity before photobleaching, measured as a function of time. The area under the curve (AUC) was calculated for each bleached cell.

### Propidium iodide uptake assay

The membrane permeability of wild type and mutant Cx30.3-expressing keratinocytes to propidium iodide (PI) was assessed as previously described with some modifications (Tong et al., 2007; Lucaciu et al., 2022). Briefly, Cx43KO REKs were seeded in six-well plates with a glass coverslip and transfected with 0.1 µg plasmid DNA encoding FLAG-tagged Cx30.3 and Cx30.3 mutants as described above. As controls, one well was untreated (denoted “control”) while another well was treated with all transfection reagents except for plasmid DNA (denoted “lipofectamine”). 24 h after transfection, the growth media was aspirated, and cells were gently washed twice with either divalent cation-free extracellular solution (DCF-ECS; 140 mM NaCl, 5.4 mM KCl, 2 mM EGTA, 10 mM HEPES, 25 mM D-Glucose, pH 7.35, osmolarity adjusted to 298 mOsm/L) or extracellular solution containing divalent cations (ECS; 140 mM NaCl, 5.4 mM KCl, 1.4 mM MgCl_2_, 2 mM CaCl_2_, 10 mM HEPES, 25 mM D-Glucose, pH 7.35, osmolarity adjusted to 298 mOsm/L). Subsequently, cells were incubated in DCF-ECS or ECS supplemented with 150 µM propidium iodide (PI; 668.5Da; ThermoFisher, Cat# P1304MP) and 2.5 μg/mL calcein, AM (ThermoFisher, Cat# C3100MP) for 15 min at 37°C and 5% CO_2_. The dye solution was then aspirated, and cells were washed gently three times with ECS. Cells were then transferred to a Zeiss LSM800 confocal microscope and imaged using a ×10 objective lens. Five random frames (∼400,000 µm^2^) were captured for each condition. The number of PI-positive cells per frame were quantified using the Cell Counter plugin (https://imagej.nih.gov/ij/plugins/cell-counter.html) for ImageJ (https://imagej.nih.gov/ij/).

Transfection efficiency was quantified in parallel for each condition using cells plated on the coverslips in each dish. Prior to dye loading, the coverslips were removed, and cells were fixed in 10% (v/v) neutral buffered formalin (VWR, Mississauga, ON, Canada, Cat# 10790-714) for 20 min at room temperature. Cells were then permeabilized with 0.1% Triton X-100 (MilliporeSigma, Cat# 9002-93-1) for 10 min at room temperature before immunolabeling for FLAG as described above. Five random fields of view (∼25,500 µm^2^) were captured using a 40× water-immersion lens and the number of total cells (identified by Hoechst 33342-positive nuclei) and FLAG-positive cells were quantified using the Cell Counter plugin for ImageJ. At least 180 total cells were quantified per coverslip to estimate transfection efficiency.

### Cell treatments

To assess the ability of sub-physiological temperature incubation to improve protein folding and trafficking, cells expressing Cx30.3 or EKVP-linked mutants were incubated at 26°C for 48 h, fixed and subsequently immunolabelled as described earlier. To assess the potential for rescue of EKVP-linked mutants with chemical chaperones, connexin or mutant expressing cells were treated with 5 mM 4-phenylbutyrate (4-PBA; MilliporeSigma, Cat# 1716-12-7), or 500 μM tauroursodeoxycholic acid (TUDCA; MilliporeSigma, Cat# 14605-22-2). Drug treated cells were fixed and immunolabelled as described earlier. Connexin or mutant expressing cells treated with 10% (v/v) glycerol Caledon Laboratories, Georgetown, ON, Canada Cat# 5350-1) for 24 and 48 h were grown in glass-bottom 35 mm dishes and live-imaged as described earlier.

### Statistical analysis

All data are shown as mean ± standard error of the mean (SEM) and analyzed with either a Mann-Whitney test for two group analysis or a one-way or two-way ANOVA test with multiple comparisons for analysis of more than two groups. Normality tests were performed for all data to determine if parametric or non-parametric analysis should be used. All statistical analysis was performed using GraphPad Prism Version 8 (GraphPad Software, San Diego, California, United States, www.graphpad.com).

## Results

### GFP-, RFP-, and FLAG-tagged Cx30.3 assembled into gap junctions in keratinocytes

**FIGURE 1:**
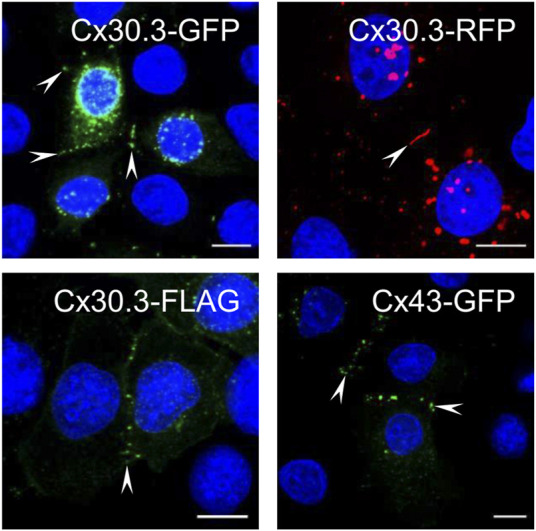
Cx30.3 traffics and assembles into gap junctions in keratinocytes. GFP- (green), RFP- (red), and FLAG-tagged (green) wild type Cx30.3 assemble into gap junction plaques (arrowheads) at cell-cell interfaces in Cx43KO REKs similar to GFP-tagged Cx43 (green). Nuclei are demarcated by Hoechst 33342 staining (blue). Scale bars = 10 μm.

Keratinocytes express multiple connexins including Cx30.3, yet surprisingly no studies have previously investigated the ability of human Cx30.3 to assemble into gap junctions when expressed in keratinocytes. Here, we show that GFP-, RFP- and FLAG-tagged Cx30.3 all assembled into gap junctions in Cx43KO REKs similar to GFP-tagged Cx43 (Figure 1, arrowheads). It is notable that GFP- and RFP-tagged Cx30.3 tended to form variable numbers of perinuclear inclusion bodies suggesting that the large fluorescent protein tags may attenuate the efficiency for Cx30.3 to form gap junctions. On the other hand, the small FLAG tag appeared to allow ectopically expressed Cx30.3 to form a greater number of gap junctions although punctate intracellular deposits of Cx30.3 were also identified in some cells. Collectively, we concluded that Cx30.3 is a gap junction forming connexin when expressed in keratinocytes in keeping with the vast majority of connexins that have been studied.

### Cx30.3 mutants occur on highly conserved residues and are predicted to be pathogenic

**FIGURE 2:**
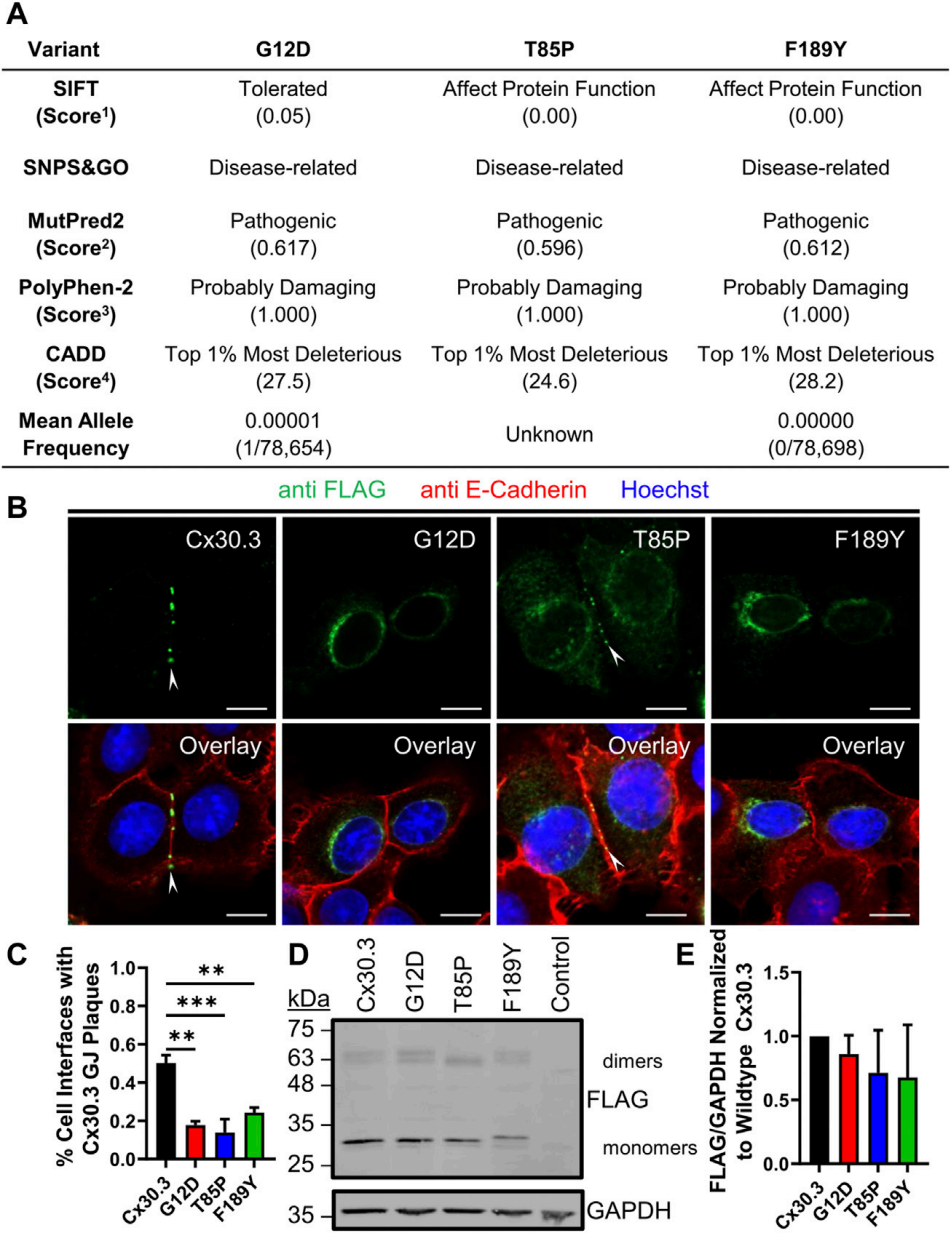
EKVP-linked Cx30.3 mutants exhibit impaired gap junction assembly compared to wild type Cx30.3. **(A)** Summary of *in silico* predictions of the pathogenicity of the G12D, T85P, and F189Y Cx30.3 mutants. Score thresholds are as follows: ^1^Score <0.05 = not tolerated, ^2^Score >0.500 = pathogenic, ^3^Score >0.850 = probably damaging. ^4^Score between 20 and 29.9 = top 1% most deleterious substitutions predicted in the human genome. **(B)** FLAG-tagged Cx30.3 mutants (green) exhibit impaired assembly into gap junction plaques (arrowheads) at cell-cell interfaces, as demarcated by the proxy marker for the plasma membrane, E-cadherin (red). **(C)** Quantification reveals that Cx43KO REK pairs expressing Cx30.3 mutants exhibit less gap junctions compared to cells expressing wild type Cx30.3 (*N* = 3). **(D)** Representative immunoblot showing relatively equal expression of FLAG-tagged Cx30.3 mutants. The band corresponding to the F189Y mutant migrated slightly slower than other mutants. **(E)** Densitometric analysis reveals relatively equal expression of Cx30.3 mutants in Cx43KO REKs (*N* = 3). Nuclei are demarcated by Hoechst 33342 staining (blue). Scale bars = 10 μm **p < 0.01; ***p < 0.001.

Bioinformatic analysis revealed that all three gene mutations were absent from the genome aggregation database (gnomAD) suggesting that the mutations that lead to G12D, T85P, and F189Y occur at extremely low frequencies and have not been detected in healthy human subjects. Multi sequence alignment analysis revealed that the G12, T85, and F189 residues are highly conserved in Cx30.3 across 86 species, in all seven human β-connexins, and in 675 β-connexins across species (Supplementary Figure S1) suggesting that there is strong natural selection pressure on these positions. In the case of T85, this position can tolerate a closely related serine residue in place of threonine, or in some cases the threonine may be in position 86 instead (Supplementary Figure S1). Subsequent *in silico* analysis of the pathogenicity of the G12D, T85P, and F189Y mutants using four different algorithms almost unanimously predicted these mutants to be pathogenic, probably damaging, or affecting protein function (Figure 2A). The only exception was the G12D mutant that was predicted to be tolerated by the SIFT algorithm, however, this mutant scored directly at the threshold of being tolerated (threshold score = 0.05) and was predicted to be pathogenic or probably damaging by all other algorithms.

### Cx30.3 mutants exhibit impaired trafficking and assembly into gap junctions in keratinocytes

To characterize the ectopic expression and cellular localization of Cx30.3 mutants, we generated and expressed FLAG-tagged Cx30.3 and EKVP mutants in REKs and counterstained the keratinocytes for the adherens junction protein E-cadherin as a proxy marker of cell-cell interfaces (Figure 2B). FLAG-tagged Cx30.3 efficiently assembled into gap junctions while Cx30.3 mutants were frequently found within intracellular compartments (Figure 2B). However, repeatedly a population of Cx30.3 mutants were found to also form a few gap junctions (Figure 2B, see T85P as an example). The ability of the FLAG-tagged Cx30.3 mutants to form gap junctions was found to be reduced by ∼50%–70% when compared to wild type Cx30.3 (Figure 2C). Western blots further revealed that none of the Cx30.3 mutants were particularly unstable or rapidly degraded as all mutants were found at comparable levels to wild type Cx30.3 (Figures 2D, E). However, the F189Y mutant exhibited slightly slower electrophoretic mobility (Figure 2D).

### EKVP-linked Cx30.3 mutants were frequently retained within the endoplasmic reticulum or Golgi apparatus and fail to form functional gap junctions

**FIGURE 3:**
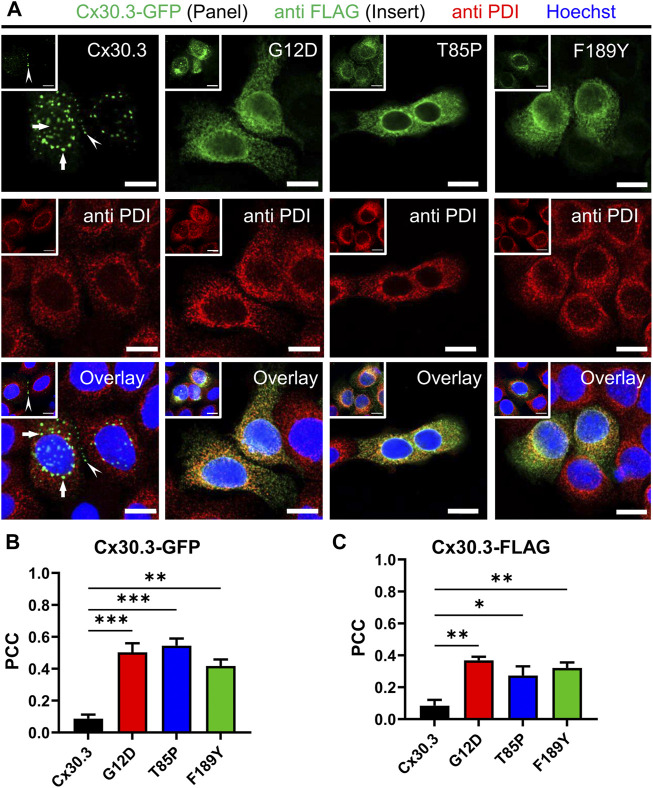
Cx30.3 mutants are localized to the endoplasmic reticulum. **(A)** GFP-tagged or FLAG-tagged (inserts) Cx30.3 (green) traffics and assembles into gap junctions (arrowheads). FLAG-tagged Cx30.3 was detected in noticeably fewer intracellular/perinuclear inclusion bodies than GFP-tagged Cx30.3 (arrows). In contrast, both GFP- and FLAG-tagged Cx30.3 mutants exhibit a primarily intracellular distribution that colocalizes with the ER resident protein PDI (red). Nuclei are demarcated by Hoechst 33342 staining (blue). Scale bars = 10 μm. Quantification by Pearson’s correlation coefficient (PCC) indicates that **(B)** GFP- and **(C)** FLAG-tagged Cx30.3 mutants significantly co-localize with PDI (*N* = 3 for Cx30.3-GFP; *N* = 4 for Cx30.3-FLAG). *p < 0.05; **p < 0.01; ***p < 0.001.

**FIGURE 4:**
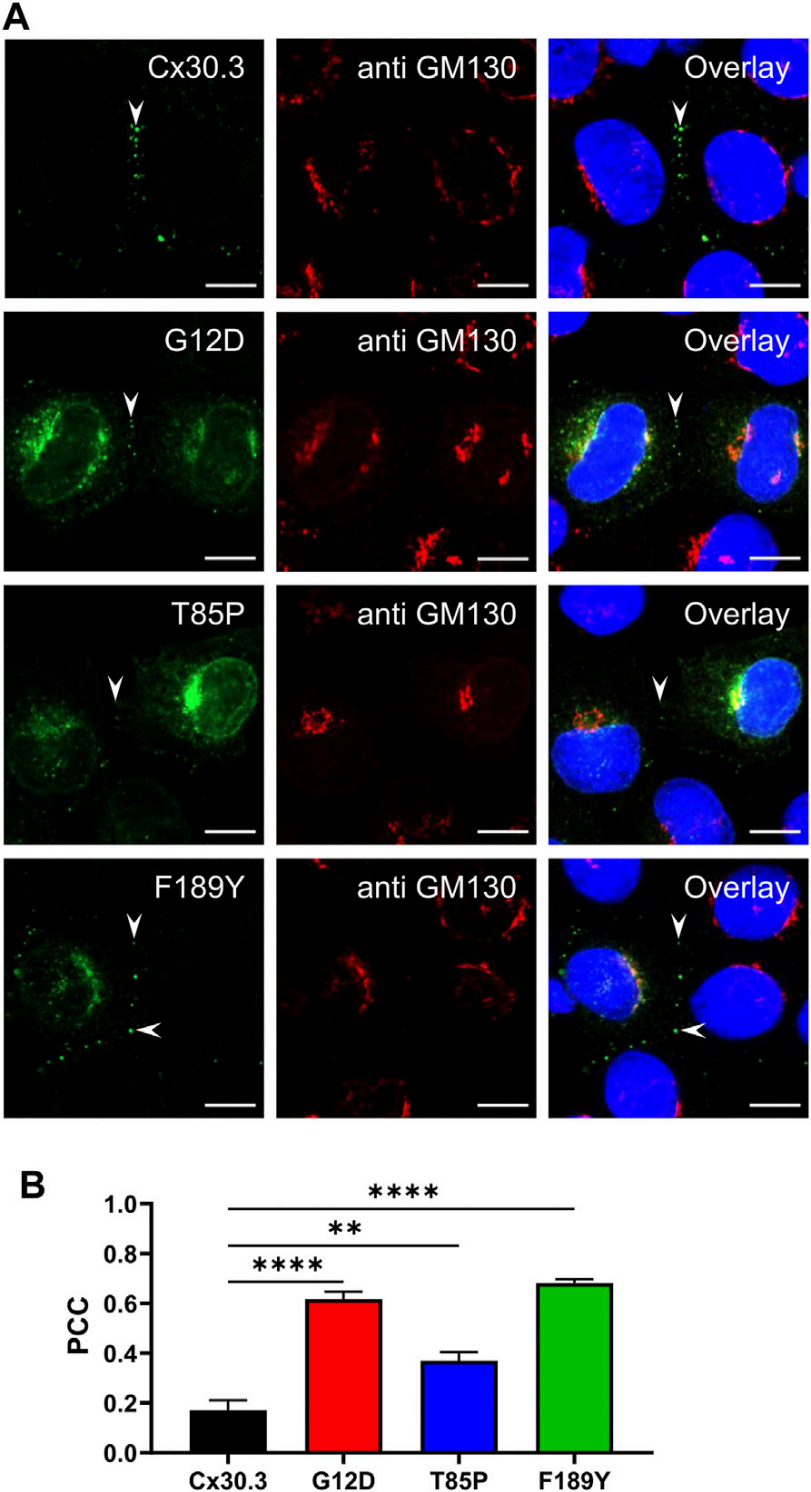
Cx30.3 mutants occasionally localize to the Golgi apparatus. **(A)** In some cells, FLAG-tagged Cx30.3 mutants (green) noticeably localize to paranuclear caps that stained positively for the Golgi apparatus resident protein GM130 (red). Gap junctions are indicated by arrowheads. Nuclei are demarcated by Hoechst 33342 staining (blue). Scale bars = 10 μm. **(B)** Quantification by PCC reveals that the Cx30.3 mutants exhibit significant co-localization with GM130 (N = 3). **p < 0.01; ****p < 0.0001.

To characterize the subcellular localization of wild type Cx30.3 and EKVP-linked mutants, Cx43-ablated REKs were genetically engineered to express GFP-tagged or FLAG-tagged Cx30.3 or EKVP-linked G12D, T85P, and F189Y Cx30.3 mutants. While wild type Cx30.3 assembled into gap junctions (or intracellular structures resembling inclusion bodies), all three mutants were found localized to a reticular network that was continuous with the nuclear envelope reminiscent of the ER (Figure 3A) or found in paranuclear locations. Further immunolabelling for the ER-lumen resident protein, PDI, revealed that both GFP- and FLAG-tagged G12D, T85P, and F189Y mutants were substantially retained in the ER (Figure 3A), a conclusion supported when the Pearson’s correlation coefficient (PCC) was calculated (Figures 3B, C). To further interrogate the paranuclear compartment housing a population of FLAG-tagged Cx30.3 mutants, mutant expressing cells were immunolabeled for the resident Golgi apparatus protein, GM130 (Figures 4A, B). Here distinct populations of all three mutants were found to localize to the Golgi apparatus. Collectively, we concluded that whether the Cx30.3 mutants were GFP- or FLAG-tagged they exhibited an impaired ability to traffic through the secretory pathway and form prototypical gap junctions, although this impairment was more pronounced when using GFP-tagged mutants.

**FIGURE 5:**
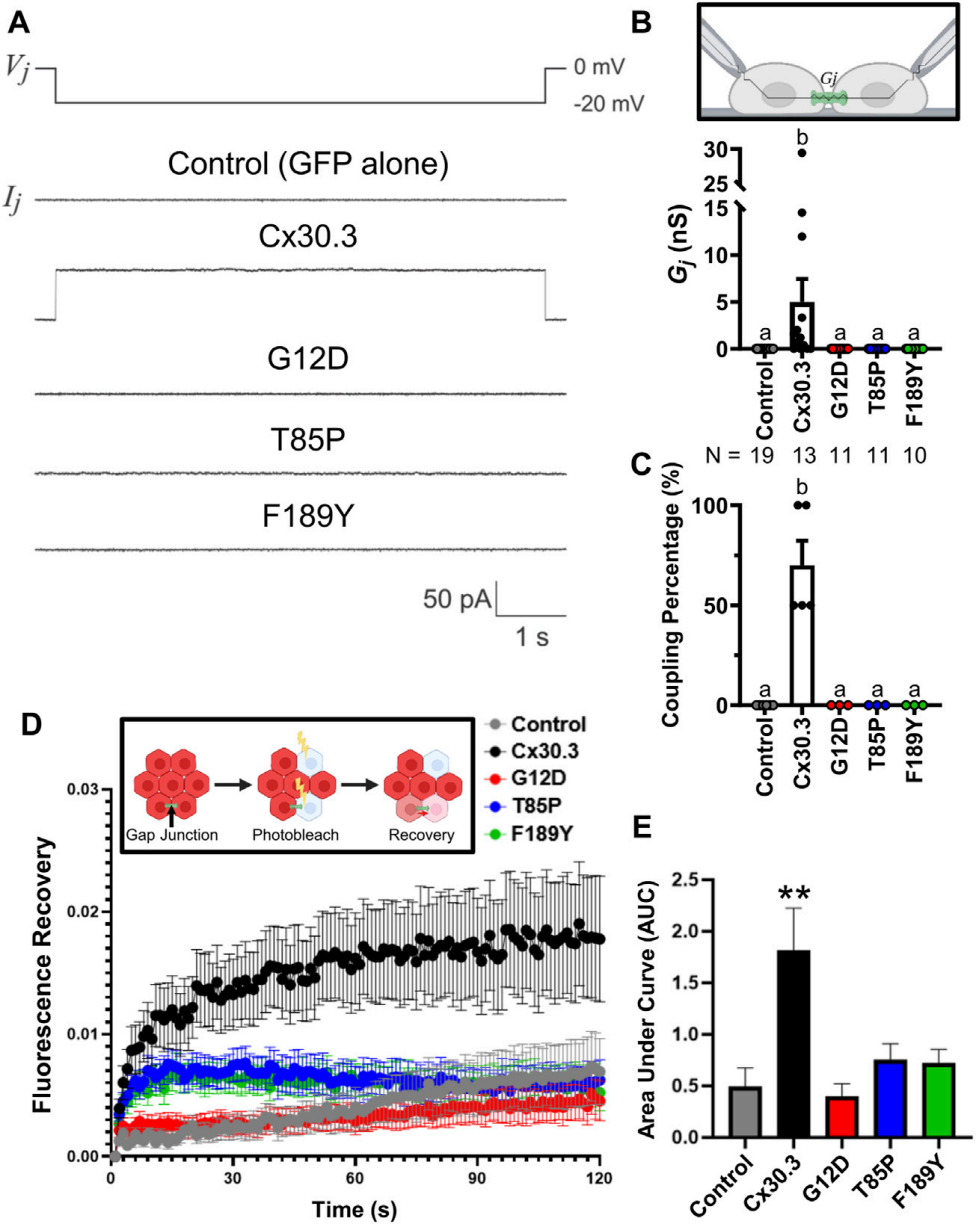
Cx30.3 mutants do not form functional gap junctions. **(A)** Representative recordings of junctional currents (*I*_*j*_) following a −20 mV trans-junctional voltage pulse (*V*_*j*_) in N2a cells expressing GFP-tagged wild type or mutant Cx30.3, or free GFP (Control). **(B)** Quantification of macroscopic junctional conductance (*G*_*j*_) indicates that N2a cells expressing wild type Cx30.3 form functional gap junctions, while control (GFP alone) and mutant-expressing cells do not. N values below the graph represents the number of cell pairs expressing each mutant analyzed. **(C)** Analysis of the coupling percentage of control N2a cells or N2a cells expressing GFP-tagged wild type or mutant Cx30.3. **(D)** Fluorescence recovery in Cx43KO REKs, mediated by junctional transfer of unbleached calcein red-orange dye from coupled cells, was monitored over 120 s. **(E)** Quantification of the area under the curve reveals that wild type Cx30.3 enhances dye transfer in Cx43KO REKs, while Cx30.3 mutants do not (*N* = 3). **p < 0.01. Groups with different letters are statistically different from groups marked with other letters (*p* < 0.05). Schematics in **(B, D)** were created using BioRender.com.

To assess if the Cx30.3 mutants could form functional gap junction channels when expressed in the absence of other connexins, GFP-tagged mutants were expressed in GJIC-deficient N2a cells. Paired cells that both expressed either wild type or Cx30.3 mutants were subject to patch-clamp electrophysiology and junctional currents were recorded (Figure 5A). Quantification of the gap junctional coupling conductance (*G*_*j*_) revealed that wild type Cx30.3 formed functional intercellular channels but all three Cx30.3 mutants and the GFP (negative control) did not (Figure 5B). Cell coupling percentage analysis revealed that ∼70% of N2a cell pairs expressing wild type Cx30.3 were electrically coupled compared to 0% of control or mutant expressing N2a cells (Figure 5C). Confirmation that the Cx30.3 mutants failed to form gap junctions beyond that observed in controls was confirmed when fluorescence recovery after photobleaching was performed on calcein red-orange loaded Cx43KO REK pairs and cell clusters that expressed wild type Cx30.3 or the various Cx30.3 mutants (Figure 5D). Area under the curve was quantified and revealed that the expression of wild type Cx30.3, but not the EKVP-linked Cx30.3 mutants, significantly increased the amount of dye recovery (Figure 5E), suggesting the mutants were not forming functional gap junction channels capable of dye transfer beyond that seen in controls.

### Cx30.3 mutants did not elevate cellular BiP/GRP78 levels

**FIGURE 6:**
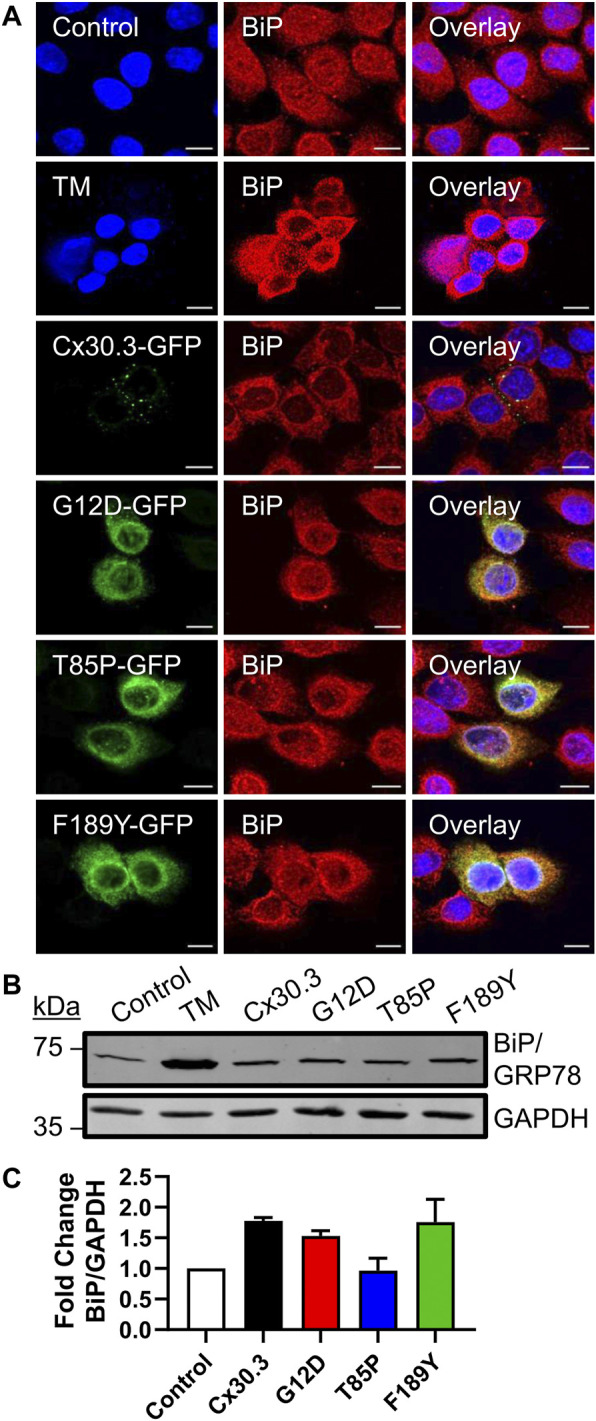
Cx30.3 mutants do not upregulate BiP/GRP78. **(A)** Representative confocal images of Cx43KO REKs expressing GFP-tagged wild type or mutant Cx30.3 (green) labeled for BiP/GRP78 (red). Cx43KO REKs treated with 2 μg/mL tunicamycin (TM) to induce ER stress were used as a positive control for BiP/GRP78 upregulation. Untreated Cx43KO REKs served as a negative control. Nuclei counterstained with Hoechst 33342 (blue). Scale bars = 10 µm. **(B)** Representative immunoblot of BiP/GRP78 levels in Cx43KO REKs expressing wild type or Cx30.3 mutants, as well as negative and positive controls. GAPDH was used as a loading control. **(C)** Densitometry quantification reveals that the expression of either wild type or mutant Cx30.3 did not result in BiP/GRP78 upregulation (*N* = 3).

Since the Cx30.3 mutants were primarily retained in the ER of REKs, we sought to determine whether BiP/GRP78 levels were elevated as one measure of ER stress. Qualitative immunofluorescence analysis revealed no apparent upregulation of BiP/GRP78 levels compared to neighbouring control cells that did not express the mutants, while BiP/GRP78 expression was clearly induced in keratinocytes treated with tunicamycin (Figure 6A). Quantitative immunoblot analysis showed no evidence of increased BiP/GRP78 levels (Figures 6B, C).

### Mutant expressing keratinocytes were more susceptible to propidium iodide uptake

**FIGURE 7:**
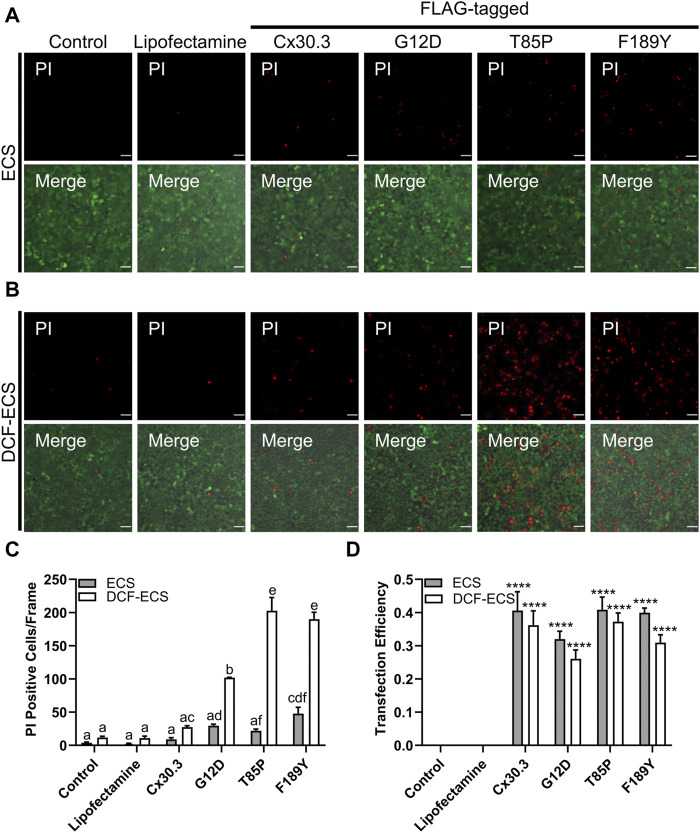
Mutant expressing keratinocytes are more permeable to propidium iodide. Representative images of Cx43KO REKs expressing FLAG-tagged wild type or Cx30.3 mutants and loaded with propidium iodide (PI; red) and calcein AM (green) in either **(A)** extracellular solution (ECS) containing the divalent cations Ca^2+^ and Mg^2+^ or **(B)** divalent cation-free extracellular solution (DCF-ECS). **(C)** Quantification reveals that significantly more cells expressing Cx30.3 mutants took up PI in the absence of divalent cations compared to both of the controls and wild type Cx30.3 in both ECS (grey bars) and DCF-ECS (white bars) conditions. Groups marked with the same letter are not significantly different from one another. **(D)** Parallel quantification of transfection efficiency reveals that a similar proportion of cells expressed Cx30.3 across all conditions. ****p < 0.0001 compared to control and lipofectamine groups in the same extracellular solution.

Since several disease-linked connexin mutants have been linked to cell death and/or hyperactive or leaky hemichannels, we evaluated the effect of the mutants on cell membrane permeability to the normally impermeant fluorescent dye propidium iodide (PI). We found that keratinocytes expressing two of the three Cx30.3 mutants exhibited a non-significant trend towards an increase in PI uptake in the presence of extracellular divalent cations (Ca^2+^ and Mg^2+^) but significance was reached for the F189Y mutant (Figures 7A, C). However, when divalent cations were removed in an attempt to potentially induce the opening of hemichannels, we found that keratinocytes expressing all Cx30.3 mutants were significantly more susceptible to PI uptake compared to controls and keratinocytes expressing wild type Cx30.3 (Figures 7B, C). These findings were independent of transfection efficiency as all plasmids were similarly expressed in REKs (Figure 7D).

### Strategies to improve ER function and protein folding failed to increase Cx30.3 mutant assembly into gap junctions

In the past, various drug and reduced-temperature approaches to improve ER function and protein folding have been employed to increase the intracellular transport and delivery of disease-linked mutants, including a cataract-linked Cx50 mutant, to the cell surface (Cortez and Sim, 2014; Uppala et al., 2017; Jara et al., 2018; Estabrooks and Brodsky, 2020) raising the notion that similar strategies may effectively increase the delivery of Cx30.3 mutants to the cell surface and their assembly into gap junctions. First, we found that incubating Cx43-ablated REKs engineered to express Cx30.3 or EKVP-linked mutants at 26°C for 48 h revealed no improvement in gap junction formation (Supplementary Figure S2). To assess the potential of chemical chaperones to rescue EKVP-linked mutants, Cx43-ablated REKs engineered to express Cx30.3 or EKVP-linked Cx30.3 mutants were treated with either 500 μM TUDCA (Supplementary Figure S3), 5 mM 4-PBA (Supplementary Figure S4), or 10% (v/v) glycerol (Supplementary Figure S5). All treatments failed to improve mutant or wild type Cx30.3 delivery to the cell surface and gap junction formation. These results suggest that conditions designed to improve ER function and protein folding were ineffective at improving the assembly of wild type Cx30.3 or Cx30.3 mutants into gap junctions.

### Wild type Cx30.3 rescues the transport of Cx30.3 mutants and their assembly into gap junctions

**FIGURE 8:**
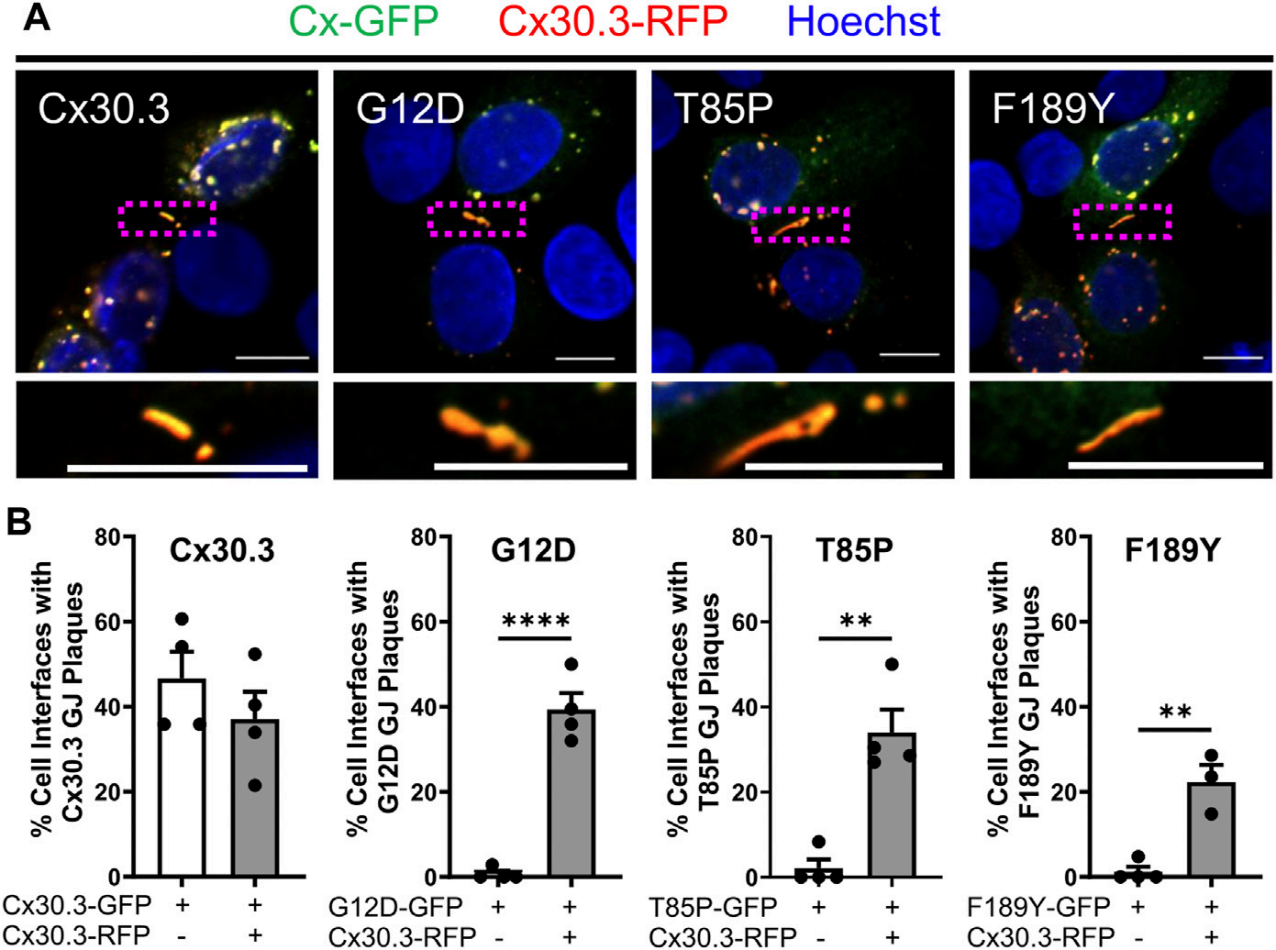
Co-expression of wild type Cx30.3 restores Cx30.3 mutant trafficking and assembly into gap junctions. **(A)** Representative images of Cx43KO REKs co-expressing GFP-tagged wild type or Cx30.3 mutants (green) and RFP-tagged wild type Cx30.3 (red). Yellow/orange signal denotes regions of overlapping red and green signals. Dashed boxes indicate the areas magnified in the inserts below. Nuclei counterstained with Hoechst 33342 (blue). Scale bars = 10 µm. **(B)** Expression of wild type Cx30.3 significantly increased the incidence of gap junctions containing Cx30.3 mutants in keratinocytes. Filled in circles (•) represent one replicate. **p < 0.01; ****p < 0.0001.

Since Cx30.3 mutants are autosomal dominant and co-expressed with wild type Cx30.3 in patients with EKVP, we wanted to assess if co-expression of wild type Cx30.3 could rescue the assembly of Cx30.3 mutants into gap junctions. Thus, we transfected REKs with equal levels of plasmids encoding GFP-tagged mutants with RFP-tagged wild type Cx30.3 and assessed gap junction plaque levels in adjacent cells expressing both Cx30.3 mutants and wild type Cx30.3 (Figure 8A). Quantification of gap junctions that contained both Cx30.3 mutants and wild type Cx30.3 revealed that all three mutants could be effectively, but differentially, rescued to the cell surface where they intermixed with wild type Cx30.3 within gap junctions (Figure 8B).

### Connexin isoforms intermix with Cx30.3 mutants and rescues their assembly into gap junctions

**FIGURE 9:**
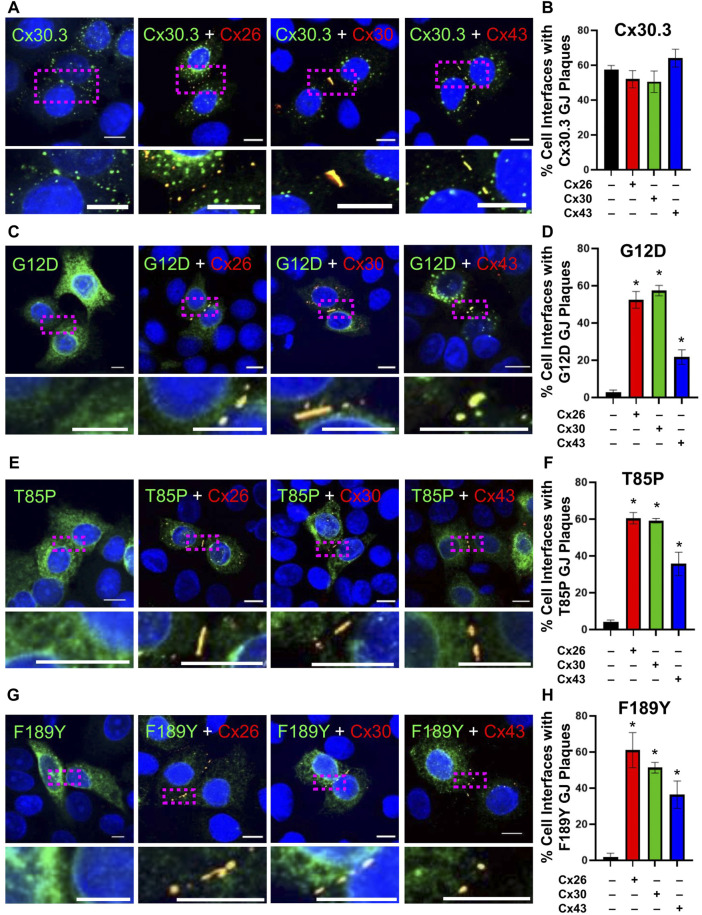
Co-expression of keratinocyte connexins partially restores Cx30.3 mutant trafficking and assembly into gap junctions. **(A, B)** Cx30.3 (green) forms gap junctions alone and when co-expressed with Cx26, Cx30, or Cx43 (red). **(C, D)** G12D (green), **(E, F)** T85P (green), and **(G, H)** F189Y (green) mutants are readily found in gap junctions when co-expressed with Cx26, Cx30, or Cx43 (red). Dashed boxes indicate the areas magnified in the inserts. Nuclei counterstained with Hoechst 33342 (blue). *N* = 3 for all experiments. Scale bars = 10 µm *p < 0.05.

Knowing that individual keratinocytes co-express multiple connexins during any stage of their differentiation within the epidermis, we investigated whether co-expressed connexins could intermix with Cx30.3 within the same gap junctions. Confocal imaging analysis revealed that not only did RFP-tagged Cx26 and Cx30 colocalize with GFP-tagged Cx30.3, but surprisingly the more sequence diverse Cx43 also intermixed with Cx30.3 when co-expressed in REKs (Figures 9A, B). Next, we addressed if these same connexin isoforms could selectively intermix with GFP-tagged Cx30.3 mutants and transdominantly rescue their assembly into gap junctions. Our findings revealed that the G12D (Figures 9C, D), T85P (Figures 9E, F) and F189Y (Figures 9G, H) could all be differentially rescued to the cell surface where they intermixed within gap junctions containing co-expressed Cx26, Cx30, and Cx43. These findings provide support to the notion that selective upregulation of keratinocyte connexins could possibly reduce the disease phenotype invoked by the Cx30.3 mutants.

## Discussion

Cx30.3 encoded by the *GJB4* gene is one of the most understudied connexins found in the epidermis. Inherited *GJB4* gene mutations are not known to cause pathologies in any organs other than the skin, even though detectable levels of this connexin have been reported in cardiomyocytes (Okamoto et al., 2020), granulosa cells (Wang et al., 2009), corneal cells (Shurman et al., 2005; Zhai et al., 2014), and airway epithelial cells (Wiszniewski et al., 2007; Kim et al., 2016). Studies using wild type and Cx30.3-deficient mice support the location and potential role for Cx30.3 in the stratum spinosum and granulosum of the epidermis, however, Cx30.3-deficient mice exhibited no skin abnormalities or changes in wound healing suggesting that co-expressed connexin isoforms may compensate for the loss of Cx30.3 to maintain a healthy epidermis (Zheng-Fischhöfer et al., 2007). In addition to serving in compensatory roles, it seems that keratinocytes differentially express multiple connexin isoforms to act as key regulators of epidermal differentiation and homeostasis (Di et al., 2001; Bedner et al., 2012; Martin et al., 2014; Faniku et al., 2015; Anderton and Alqudah, 2022). Keratinization disorders, such as EKVP, may arise when inherited gene mutations dysregulate this differentiation process (Lilly et al., 2016; Cocozzelli and White, 2019). In humans, fourteen mutations to *GJB4* encoding thirteen missense mutant proteins have been clinically linked to EKVP (Macari et al., 2000; Richard et al., 2003; Common et al., 2005; Van Steensel et al., 2009; Scott et al., 2011; Kokotas et al., 2012; Liu et al., 2012; Sbidian et al., 2013; Ishida-Yamamoto, 2016; Yoshikata-Isokawa et al., 2016; Dai et al., 2020; Zhang et al., 2021) demonstrating an important role for Cx30.3 in the human epidermis. Further, the G12D, T85P, and F189Y Cx30.3 mutants studied herein co-segregate with EKVP as they were detected in patients with a family history of EKVP going back generations highlighting the fact that *GJB4* (Cx30.3) mutations are likely causal of skin pathology in these patients (Richard et al., 2003; Van Steensel et al., 2009).

In the present study, the G12D, T85P and F189Y Cx30.3 mutants associated with EKVP were found to exhibit impaired trafficking resulting in retention within the ER and Golgi apparatus when expressed in the absence of other connexins, a finding that is consistent with the predicted deleterious outcomes of the mutants *in silico*. Despite this, all three mutants failed to activate one or more arms of the UPR as evidenced by normal levels of BiP/GRP78 expression indicating that the mutant proteins may pass or evade quality control mechanisms. Functional studies also revealed that the GFP-tagged Cx30.3 mutants were incapable of forming functional intercellular channels when expressed alone in keeping with the impaired trafficking of the mutants into optically detectable gap junctions. However, Cx30.3 mutant-expressing keratinocytes were found to be more permeable to PI in the absence of extracellular divalent cations, which may reflect disruption of the cell membrane integrity leading to cell death. Treatments designed to improve protein folding and/or ER function were ineffective in enhancing the assembly of EKVP-linked mutants into gap junctions, but the co-expression of select wild type keratinocyte connexins, including wild type Cx30.3, rescued the assembly of Cx30.3 mutants into gap junctions. These latter findings suggest that the mutants are not in a permanent misfolded or trafficking defective state and can pass or bypass quality control mechanisms associated with the secretory pathway under some circumstances.

Prior to the current study, our understanding of the role and function of Cx30.3 in cells and, particularly in keratinocytes, was limited to evidence that Cx30.3 can form gap junctions when expressed in HeLa cells (Plantard et al., 2003; Zhang et al., 2021). We found that fluorescent protein-tagged and FLAG-tagged Cx30.3 formed gap junctions in keratinocytes albeit the efficiency of gap junction formation was attenuated by the much larger fluorescent protein tag; a situation not commonly observed when GFP is tagged to other connexin isoforms (McLachlan et al., 2005; Thomas et al., 2005; 2007; Gong et al., 2006; Ableser et al., 2014; Berger et al., 2014; Kelly et al., 2015a; Au et al., 2020; Beach et al., 2020; Lucaciu et al., 2022). Consistent with previous rodent Cx30.3 studies (Hennemann et al., 1992; White and Bruzzone, 1996; Yeager and Nicholson, 2000; Bai et al., 2018), human Cx30.3 was found to be GJIC-competent as evidenced by its ability to electrically couple N2a cells and modestly pass a 570 Da calcein red-orange dye between connexin-deficient keratinocytes. Thus, we concluded that wild type human Cx30.3 successfully assembled into functional gap junctions in REKs, and these cells were a suitable keratinocyte model to interrogate the trafficking and functional status of EKVP-linked Cx30.3 mutants when expressed alone or in combination with other keratinocyte connexins.

In stark contrast to wild type Cx30.3, high-resolution confocal microscopy revealed that the G12D, T85P, and F189Y mutants all surprisingly exhibited varying degrees of trafficking impairment when expressed in keratinocytes resulting in their localization to the ER and Golgi apparatus. This localization pattern was similar to that found in HeLa cells engineered to express the EKVP-linked GFP-tagged Cx30.3 V37M mutant (Zhang et al., 2021) and NEB1 keratinocytes that expressed the EKVP-linked G12D, G12R, R42P, and C86S mutations of a second keratinocyte connexin isoform, Cx31 (Di et al., 2002). These findings lend support to the notion that connexin gene mutations that lead to trafficking deficiencies is at the root or contributes to some forms of EKVP.

The G12D, T85P, and F189Y amino acid substitutions are localized within the amino terminus, second transmembrane, and fourth transmembrane domains, respectively (Richard et al., 2003; Ishida-Yamamoto, 2016), raising questions as to why all three mutants would exhibit trafficking deficiencies. Crystal structure analysis of Cx26, which is highly homologous to Cx30.3, suggests mutations to these highly conserved domains may lead to changes in tertiary structure–especially since the G12, T85, and F189 residues are conserved across all β-connexins in humans (Maeda et al., 2009; Bai, 2016). Possible changes to domain flexibility due to the loss of a flexible glycine residue in the G12D mutant or the introduction of a kink due to the rigid proline residue in the T85P mutant could impact folding or channel gating. Indeed, EKVP-linked Cx31 G12D was shown to misfold and activate ER stress pathways downstream of IRE1α (Tang et al., 2015). The F189Y amino acid substitution introduces a tyrosine residue that is a potential phosphorylation or sulfation site, although it is not clear if this residue is accessible to enzymatic modification as it is predicted to reside within the fourth transmembrane domain, but if so, this could possibly explain its slightly slower migration on western blots. This slight change in electrophoretic mobility may also be due to a new post-translational modification site that is unveiled elsewhere in the polypeptide due to changes in F189Y mutant protein folding. Alternatively, this slower migration may be due to a change in protein interaction with SDS during electrophoresis. In the case of the T85P mutant, since the second transmembrane domain contains a highly conserved proline residue nearby at position 87, we propose the alpha helical structure of this domain would be disrupted by the introduction of a second proline residue. Indeed, these conserved regions of the Cx30.3 polypeptide are susceptible to mutations as single amino acid substitutions to the corresponding and neighbouring residues in Cx26, Cx30, Cx31, and Cx32 have also been linked to various human pathologies affecting the skin and nervous system (Common et al., 2005; Sbidian et al., 2013; Tang et al., 2015; Ishida-Yamamoto, 2016; Dai et al., 2020; Bailey et al., 2021). These putative structural changes may be at the root of why Cx30.3 mutants have difficulty passing quality control, *via* mechanisms that appear to evade the central regulator of the UPR, BiP/GRP78 (Almanza et al., 2019; Hetz et al., 2020), as well as impair gap junction assembly. Nevertheless, detailed investigations of all three branches of the UPR and other cellular stress responses are required to effectively rule-out cell stress (Duennwald, 2015).

Currently, there are no cures or specific treatments that target the pathogenic mechanisms underlying EKVP. This raises the question as to whether the trafficking impaired Cx30.3 mutants in this study could be rescued to potentially contribute to GJIC and mitigate disease severity. Therapeutic strategies employing chemical chaperones have met with some success for the treatment of disorders caused by impaired protein folding and trafficking (Chaudhuri and Paul, 2006; Calamini et al., 2012; Cortez and Sim, 2014; Estabrooks and Brodsky, 2020). To that end, 4-PBA and a sub-physiological temperature treatment partially restored the assembly of a trafficking defective Cx50 mutant into gap junctions in a pre-clinical cellular setting (Jara et al., 2018). However, these treatments, as well as TUDCA and glycerol, failed to rescue the delivery of any Cx30.3 mutants to the cell surface and their assembly into gap junctions. Thus, we posit that the Cx30.3 mutants enter a misfolded state that does not benefit from increasing the time and opportunity to fold correctly even with the assistance of chemical chaperones.

Cell death and hemichannel gain-of-function mechanisms, which are intimately linked processes, have been reported for other disease-linked connexin mutants (Delmar et al., 2018; Cocozzelli and White, 2019). Thus, we evaluated the membrane permeability of Cx30.3 mutant-expressing keratinocytes to the normally membrane impermeant dye propidium iodide. Little differences in PI uptake were detected in cells maintained under physiological condition; however, Cx30.3 mutant expressing keratinocytes were more permeable to PI when divalent cations were absent. These findings could reflect the formation of hyperactive mutant hemichannels or an unknown mechanism where the mutants reduce the integrity of the plasma membrane. The pathological opening of hemichannels has been implicated in inflammatory diseases through the release of damage-associated molecular patterns (DAMPs) that activate immune responses, involving ATP, HMGB1 and S100 proteins (Peng et al., 2022). The increased membrane permeability to PI that we observed, either due to cell death or inducible hemichannels, may facilitate the release of DAMPs which activate inflammatory responses that result in the erythema found in patients with EKVP. Indeed, pharmacological and targeted inhibition of connexin hemichannels has been shown to alleviate skin disease in mouse models of both KIDS and Clouston syndrome (Bruzzone and White, 2020; Kuang et al., 2020; Sellitto et al., 2021).

Given all three mutants examined in this study exhibit an autosomal dominant mode of inheritance, it was important to characterize the consequences of co-expressing the mutants together with wild type Cx30.3. Somewhat surprisingly we found that wild type Cx30.3 rescued the assembly of Cx30.3 mutants into gap junctions. These findings are encouraging from a clinical point of view as they act as proof-of-principle that any misfolding of the Cx30.3 mutants that occurs can be overcome by the presence of a sufficient level of wild type Cx30.3. While efforts were made to co-express equal levels of mutant and wild type Cx30.3 in keratinocytes it is not possible to discern if this was achieved, but the partial rescue of the assembly of Cx30.3 mutants into gap junctions would suggest that their pathological effects extend beyond mechanisms that are strictly linked to their trafficking and gap junction assembly deficiencies.

Since keratinocytes simultaneously express two or more connexin isoforms we also asked the question whether co-expression of other keratinocyte connexins would rescue the assembly of Cx30.3 mutants into gap junctions. We saw a similar rescue of gap junction assembly when Cx30.3 mutants were co-expressed with Cx26 and Cx30, both of which are β-connexins found within the same epidermal strata in human skin as Cx30.3 and plausibly form mixed oligomers with Cx30.3 (Di et al., 2001; Bailey et al., 2021). We also surprisingly found that the co-expression of the α-connexin Cx43 could restore the gap junction assembly of Cx30.3 mutants, although this appeared to be less effective possibly due to the sequence divergence between α- and β-connexins (Meşe et al., 2007; Bailey et al., 2021). Evidence does suggest that Cx30.3 and Cx43 co-immunoprecipitate and may localize to the same gap junctions in diseased hearts and in induced pluripotent stem cell-derived cardiomyocytes (Okamoto et al., 2020) suggesting that they may have the capacity to interact within the same connexon. Further work has also shown that rodent Cx30.3 and Cx43 form functional homomeric heterotypic intercellular channels (White and Bruzzone, 1996; Yeager and Nicholson, 2000; Bai et al., 2018). Although we showed that expression of keratinocyte connexins could restore the assembly of Cx30.3 mutants into gap junctions in keratinocytes it remains unclear if this occurs in human epidermis where these connexins may be simultaneously expressed in the same keratinocytes. Furthermore, it remains unclear whether human Cx30.3 mutants form functional homomeric or heteromeric channels when intermixed with the other wild type connexins or wild type Cx30.3 as it was not possible to separate the contribution to GJIC by the wild type connexin from that of the mutant. Further, we were not able to rule-out the possibility of haploinsufficiency, which would require an animal model of heterozygous *GJB4* deletion to study.

In summary, we have shown that three GFP and/or FLAG-tagged EKVP-linked Cx30.3 mutants exhibit trafficking deficiencies and an inability to form functional gap junctions when expressed alone with no indication that the keratinocytes engineered to express the mutants are undergoing ER stress. This trafficking impairment is an unreported mechanism that may partially underpin EKVP thus expanding the breadth of pathogenic mechanisms beyond transdominant disruption of other co-expressed connexins (Plantard et al., 2003), induction of ER stress and/or cell death (Tang et al., 2015) (He et al., 2005; Tattersall et al., 2009; Easton et al., 2018), the establishment of hyperactive hemichannels (Srinivas et al., 2019), and perturbed connexin turnover (Lucaciu et al., 2022). Based on our PI uptake studies, it is possible that EKVP patients harboring these mutants suffer from a mechanism that includes, not only a Cx30.3 mutant trafficking deficiency, but also impaired membrane permeability and activation of a cell death pathway possibly linked to hyperactive gain-of-function hemichannels. Individual connexin mutants may therefore simultaneously exhibit both gain- and loss-of-function mechanisms, highlighting the molecular complexity of EKVP. Successful rescue of these trafficking impairments *via* the co-expression of wild type connexins provides hope that therapeutics can be identified, designed or developed that drive the expression of keratinocyte connexins with the promise of mitigating or curing EKVP as current treatments are non-specific, toxic, exhibit side effects and/or have limited efficacy (Shizukawa et al., 2015; Ishida-Yamamoto, 2016; Okuda et al., 2020).

## Data Availability

The original contributions presented in the study are included in the article/Supplementary Material, further inquiries can be directed to the corresponding author.
