# Supplementary figures and images for: GJB4 variants linked to skin disease exhibit a trafficking deficiency en route to gap junction formation that can be restored by co-expression of select connexins
Source: Front Cell Dev Biol. 2023 Feb 13;11:1073805. doi: 10.3389/fcell.2023.1073805 (PMC9968944; doi:10.3389/fcell.2023.1073805)

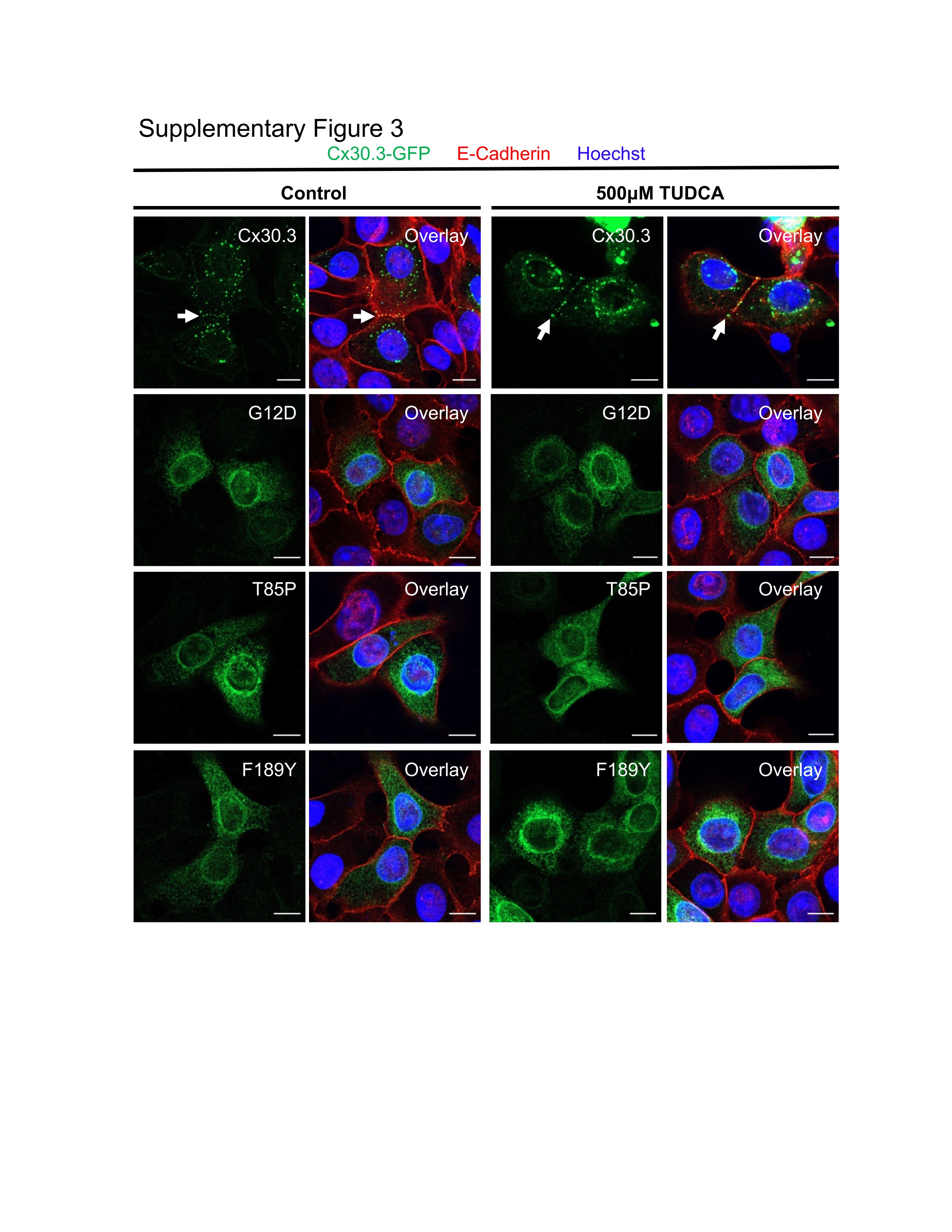

Supplement: Supplementary file 1 [file Image3.JPEG]

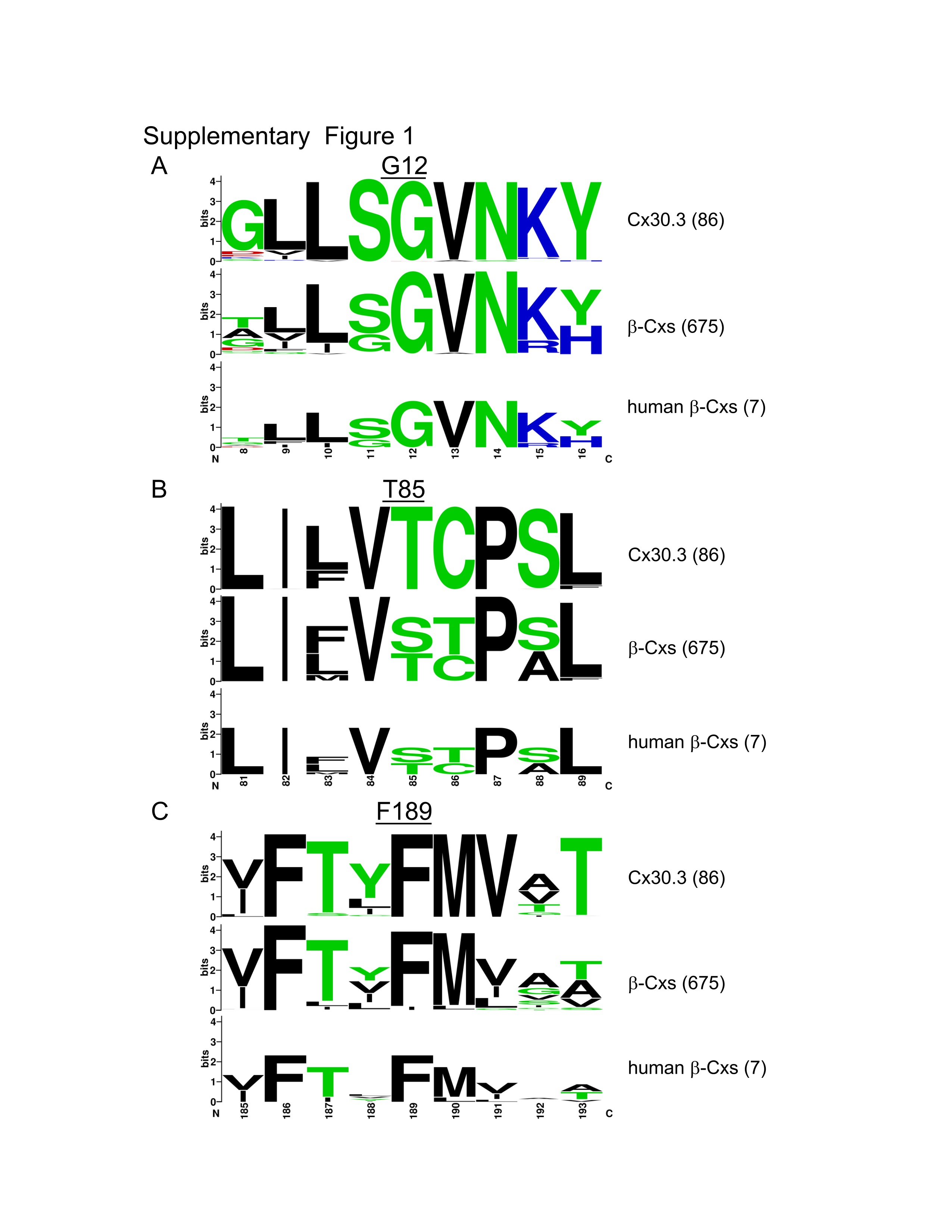

Supplement: Supplementary file 2 [file Image1.JPEG]

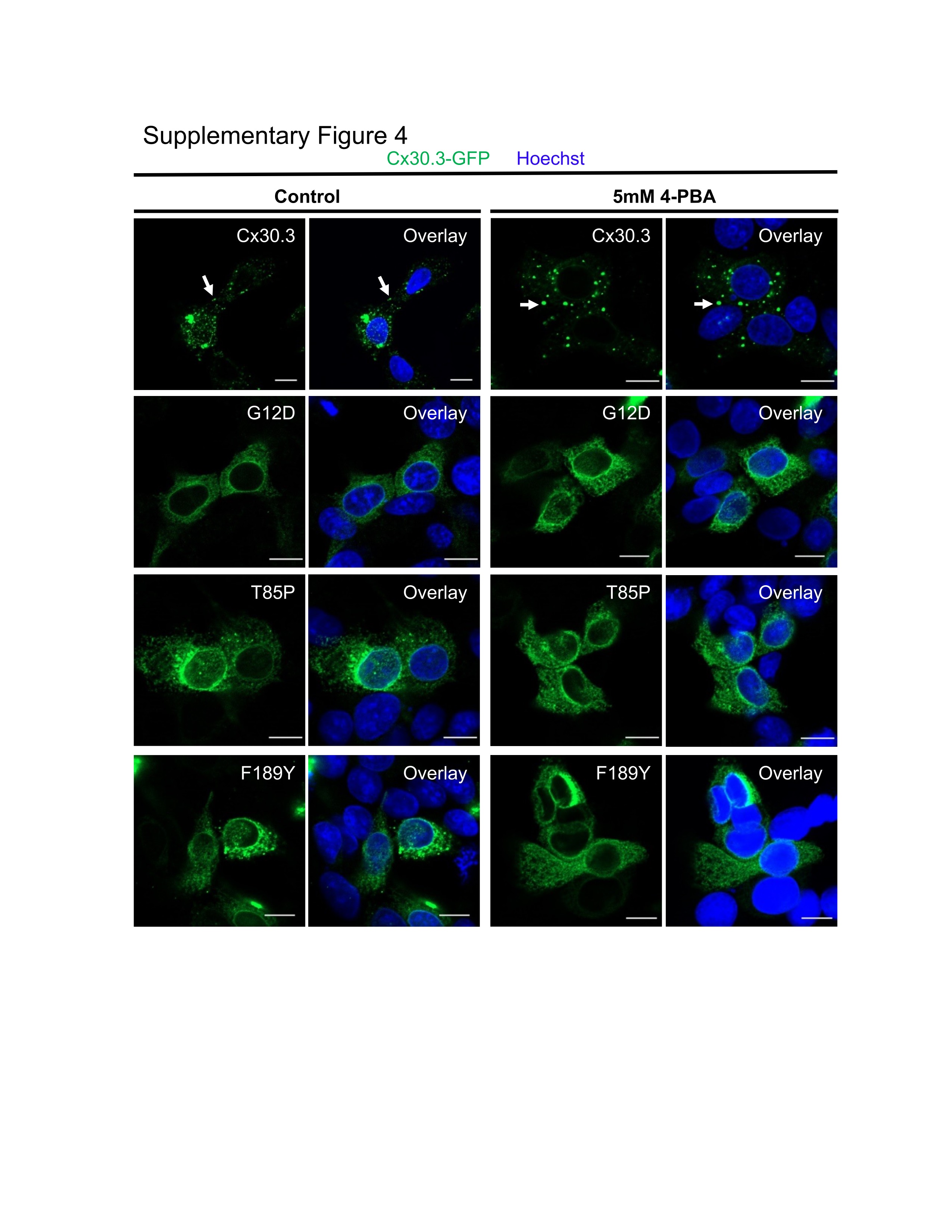

Supplement: Supplementary file 3 [file Image4.JPEG]

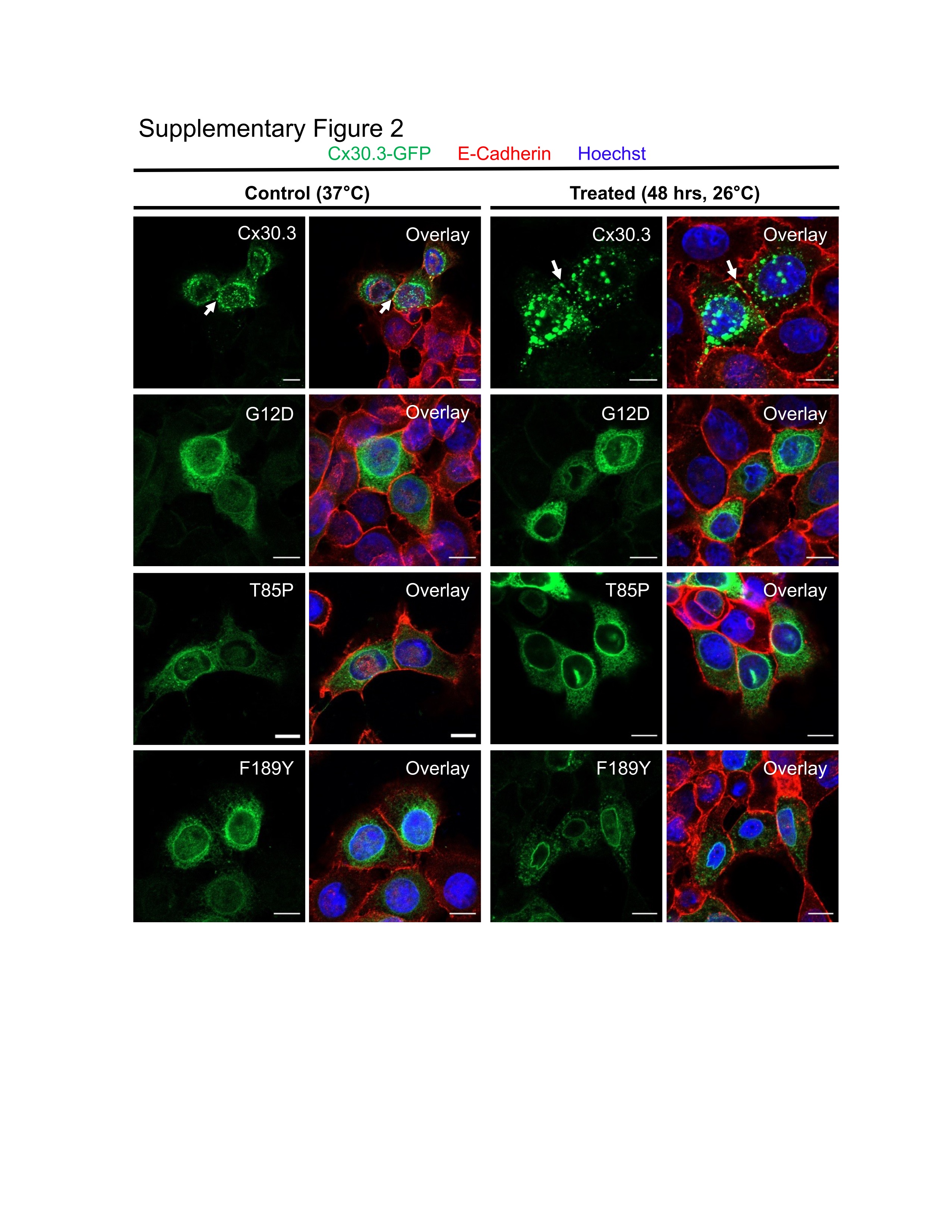

Supplement: Supplementary file 4 [file Image2.JPEG]

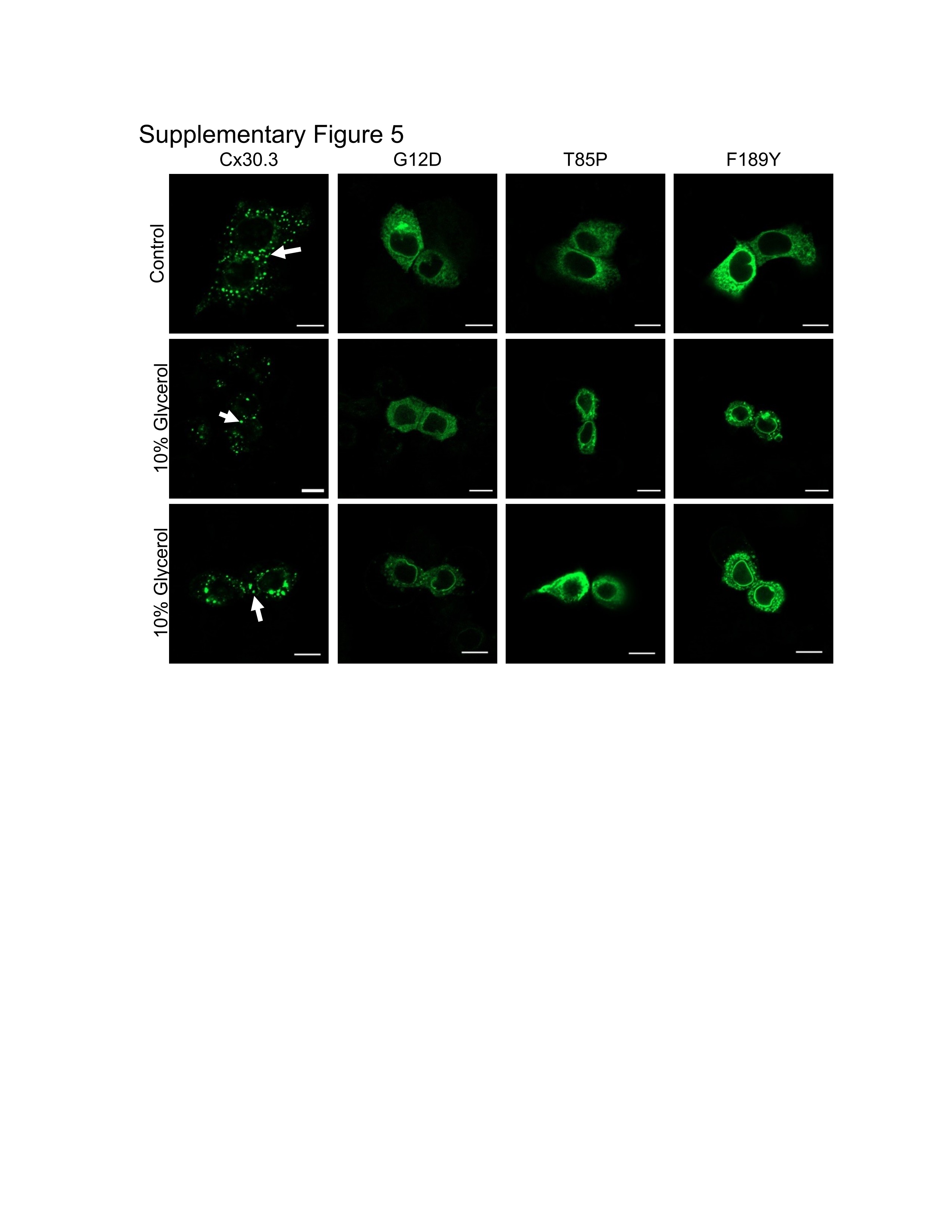

Supplement: Supplementary file 5 [file Image5.JPEG]
