# Supplementary material for: GJB4 variants linked to skin disease exhibit a trafficking deficiency en route to gap junction formation that can be restored by co-expression of select connexins
Source: Front Cell Dev Biol. 2023 Feb 13;11:1073805. doi: 10.3389/fcell.2023.1073805 (PMC9968944; doi:10.3389/fcell.2023.1073805)
Supplement: Supplementary file 6 [file DataSheet1.docx]

**Supplementary Figure Legends**

**Supplementary Figure 1. The G12, T85, and F189 residues are highly conserved in β-connexins.** Sequence logos of the Cx30.3 primary sequence show that the (a) G12, (b) T85, and (c) F189 sites of amino acid substitution linked to EKVP are highly conserved in Cx30.3 across 86 different species. Sequence logos obtained from the indicated numbers of β-connexins and all seven human β-connexins are also shown. Sequences were downloaded from <http://omabrowser.org/> and aligned using Clustal Omega. Sequence logos were prepared using <https://weblogo.berkeley.edu/logo.cgi/>. Amino acids are colored according to their chemical properties as follows: polar amino acids are colored green (G, S, T, Y, C, Q, and N), basic amino acids are colored blue (K, R, and H), acidic amino acids are colored red (D and E), and hydrophobic amino acids are colored black (A, V, L, I, P, W, F, and M).

**Supplementary Figure 2. Sub-physiological temperatures failed to restore the trafficking and the assembly of Cx30.3 mutants into gap junctions.** Wild type or mutant Cx30.3 (green)-expressing keratinocytes were incubated for 48 hours at either 37°C (control) or 26°C (treated). Microscopic analysis revealed no noticeable increase in gap junctions containing Cx30.3 mutants at cell-cell interfaces denoted by the location of E-cadherin (red). Nuclei were counterstained with Hoechst 33342. Scale bars = 10µm. Arrows indicate gap junctions.

**Supplementary Figure 3. TUDCA did not restore the trafficking and assembly of Cx30.3 mutants into gap junctions.** Wild type or mutant Cx30.3-expressing keratinocytes were incubated for 24 hours in the presence of 500 µM TUDCA or vehicle control. Confocal microscopy revealed that TUDCA treatment did not improve the assembly of Cx30.3 mutants into gap junctions at cell-cell interfaces denoted by the location of E-cadherin (red). Nuclei were counterstained with Hoechst 33342. Scale bars = 10µm. Arrows indicate gap junctions.

**Supplementary Figure 4. 4-PBA treatment failed to increase the assembly of Cx30.3 mutants into gap junctions.** Wild type or mutant Cx30.3 (green)-expressing keratinocytes were incubated for 24 hours in the presence of 5 mM 4-PBA or vehicle control. Confocal microscopy revealed that 4-PBA treatment did not improve the assembly of Cx30.3 mutants into gap junctions. Nuclei were counterstained with Hoechst 33342. Scale bars = 10µm. Arrows indicate gap junctions.

**Supplementary Figure 5. Glycerol treatment failed to improve the trafficking and assembly of Cx30.3 mutants into gap junctions.** Keratinocytes expressing wild type or mutant Cx30.3 (green) were treated with 10% (v/v) glycerol for 24 and 48 hours. Glycerol treatment failed to improve the trafficking of Cx30.3 mutants to the cell surface and assembly into gap junctions. Nuclei were counterstained with Hoechst 33342. Scale bars = 10µm. Arrows indicate gap junctions.
